# Teaching and psychological associations of adolescent piano composition skills: a study on constructivist practices and reflective guidance based on teacher reports

**DOI:** 10.3389/fpsyg.2026.1963250

**Published:** 2026-10-16

**Authors:** Xiaodong Yan

**Affiliations:** The Conservatory of Music, Northwest Normal University, Lanzhou, China

**Keywords:** adolescent piano teaching, constructivist practices, creative self-efficacy, intrinsic motivation, piano composition skills, reflective guidance

## Abstract

**Introduction:**

To understand the connection between open teaching environments and musical creative performance, this study focuses on adolescent piano teaching, examining the structural associations among constructivist practices, reflective guidance, and piano composition skills, and analyzing the parallel indirect associations linked via creative self-efficacy and intrinsic motivation for composition.

**Methods:**

A cross-sectional questionnaire design was adopted, based on the analysis of 723 self-reports from teachers with adolescent piano teaching experience and their evaluation data on students’ states and skills. Multivariate regression and Bootstrap resampling methods were used for statistical path testing, comparing group distribution characteristics under different learning purposes, and testing the boundary conditions formed by the frequency of composition teaching.

**Results:**

(1) Both constructivist practices and reflective guidance showed significant positive associations with the evaluated piano composition skills; (2) Creative self-efficacy and intrinsic motivation for composition presented significant parallel indirect paths in the above associations; (3) The overall differences among groups with different learning purposes on all core constructs reached statistical significance, with the descriptive means of the grading test or competition group generally being lower than those of the hobby group; (4) No significant moderating phenomenon of composition teaching frequency on the association between constructivist practices and intrinsic motivation was found.

**Discussion:**

Autonomous decision-making and process scrutiny are independently and positively associated with teacher-evaluated student composition skills, respectively. These teaching practice characteristics, together with students’ capability beliefs and internal willingness to participate, constitute the relevant conditions for musical practice. However, as these internal psychological states are inferred by teachers for whole groups rather than evaluated at the individual level, interpretations regarding direct psychological mechanisms remain limited. In teaching applications, compared with simply increasing the frequency of activities, improving the design quality of open and reflective tasks may be of more practical significance.

## 1 Introduction

Piano education has long emphasized performance technique, sight-reading, interpretation of works, and the degree of repertoire completion. Teaching based mainly on teacher demonstration, student imitation, and repetitive practice helps in the training of basic performance skills. However, in improvisation and composition situations, relying solely on predetermined answers and standardized requirements may constrain students’ independent exploration of melody, harmony, texture, and musical form. Adolescents are in a stage of continuous development of cognitive abilities, aesthetic judgment, and self-concept. Their piano learning involves not only motor skills and musical knowledge but also idea generation, scheme selection, work revision, and the formation of expressive intentions (Burnard, 2012; Webster, 2016). Core works on children’s and adolescents’ composition pedagogy emphasize the importance of creative thinking in music (Wiggins, 2015; Hickey, 2012) and learning through active, contextualized music-making (Kaschub and Smith, 2009; Barrett, 2012). Therefore, it is necessary to examine the relevant factors of adolescent piano composition skills from the perspective of combining teaching practices with learning psychology.

The constructivist view of learning posits that learners form an understanding of knowledge within existing experiences, social interactions, and specific contexts. In piano composition teaching, constructivist practices emphasize students’ participation in composition decision-making and the application of musical knowledge in specific tasks; teachers mainly assume the responsibilities of task design, process support, and feedback guidance (Taylor et al., 1997). Compared with standard-answer-oriented teaching, this approach focuses more on students’ autonomous participation and meaning construction. Existing research has discussed the relationship between learner-centered teaching and musical creative activities, but quantitative research on adolescent piano improvisation and composition is still limited, with most existing evidence deriving from classroom cases, teaching experience summaries, or small-scale interventions.

Reflective guidance is another teaching element in composition learning. Musical composition usually involves cyclical activities such as conceptualizing, sight-reading, listening, evaluating, and revising. Teachers asking students to explain the reasons for their handling of harmony, texture, or pedals, reviewing the more satisfying parts and those needing adjustment in their works, or participating in peer assessments can help students transform implicit auditory judgments into expressible and discussable experiences (Kember et al., 2000). Constructivist practices and reflective guidance are connected but have different focuses: the former focuses on the learning context, autonomy, and the way knowledge is constructed, while the latter focuses on the scrutiny of the composition process and its outcomes. Including both in the same analytical framework can examine their respective associations with piano composition skills.

The relationship between teaching practices and composition skills may also involve students’ psychological states (Zelenak, 2024). Creative self-efficacy reflects students’ judgments of their ability to complete creative tasks (Tierney and Farmer, 2002), while intrinsic motivation for composition involves their tendency to participate in composition based on interest and the satisfaction of the activity itself. Music education research rarely examines the parallel indirect relationships presented by both in the same model.

Adolescent piano learning is also related to teaching settings, learning objectives, and teachers’ teaching backgrounds. Different settings may have differences in curriculum arrangements, evaluation methods, and teaching resources. Particularly, an orientation toward grading tests or competitions may also correspond to a compressed classroom space for composition and improvisation activities (Hickey, 2012; Burnard, 2012). Incorporating these background factors into the analysis as contextual variables helps to examine the performance of core variable relationships under different teaching contexts.

Based on this, this study takes teachers with adolescent piano teaching experience as the survey subjects to examine the structural associations among constructivist practices, reflective guidance, teacher-perceived student creative self-efficacy and intrinsic motivation for composition, and teacher-evaluated student piano composition skills, and analyzes the group characteristics under different learning purposes. The study focuses on three questions: Are constructivist practices and reflective guidance, respectively, related to composition skills? Do creative self-efficacy and intrinsic motivation for composition present parallel indirect associations? Does composition teaching frequency constitute a boundary condition for the relationship between CP and IM?

## 2 Theoretical framework and research hypotheses

Integrating sociocultural theory, self-efficacy theory, and self-determination theory, this study constructs an association model among teaching practices, students’ psychological states, and teacher-evaluated piano composition skills.

### 2.1 Association between teaching practices and piano composition skills

Vygotsky’s sociocultural theory posits that individuals’ cognitive development occurs within social interactions and cultural activities (Vygotsky and Cole, 1978). Learners participate in activities through language, symbols, and tools, and form an understanding of knowledge in interactions with teachers and peers. In music education, this perspective emphasizes that children develop musical agency and understanding through active participation in communities of musical practice (Barrett, 2012; Wiggins, 2015). Teachers provide support adapted to learners’ current capabilities in this process, enabling them to handle tasks that they could not yet complete independently based solely on their existing abilities. This theory understands knowledge as cognitive structures formed in specific social contexts, rather than fixed content unilaterally transmitted from teachers to students.

Piano composition involves auditory imagination, application of musical knowledge, practical action, and aesthetic judgment. Students need to generate musical materials, compare different schemes, and modify works according to expressive intentions. Constructivist practices allow students to choose themes, styles, and expression methods within an appropriate range, applying music theory knowledge to the composition problems being handled. Teachers provide scaffolding through task design, questioning, and feedback, while reserving space for students to make judgments about their works (Meissner, 2021). Because fluency, novelty, structural integrity, and aesthetic expression in composition skills all involve the flexible use of knowledge, teacher-reported constructivist practices may be positively related to their evaluation of students’ composition skills.

Reflective guidance focuses on how students scrutinize the composition process and its outcomes. Describing auditory feelings, explaining musical treatment, comparing work versions, or participating in peer evaluation after sight-reading requires students to transform immediate experiences into expressible and testable judgments. Such activities do not aim for teachers to make decisions for students, but prompt them to explain, examine, and revise their choices, and thus may be related to musical material organization, work revision, and expressive consistency. Based on the above discussion, this study proposes:

*H1*: Constructivist practices (CP) are positively associated with teacher-perceived adolescent piano composition skills (CSC).

*H2*: Reflective guidance (RG) is positively associated with teacher-perceived adolescent piano composition skills (CSC).

### 2.2 Parallel indirect relationships of creative self-efficacy and intrinsic motivation for composition

The relationship between teaching practices and composition skills is not necessarily only directly associated. Students’ creative ability beliefs and motivational states manifested in classroom interactions may constitute psychological links connecting teaching practices and skill evaluations. Based on self-efficacy theory and self-determination theory (Deci and Ryan, 2000), respectively, this study incorporates creative self-efficacy and intrinsic motivation for composition into a parallel indirect paths model, examined practically through the lens of teachers’ evaluations of their students’ collective behavioral manifestations.

Self-efficacy theory defines self-efficacy as an individual’s judgment of their capability to organize and execute specific actions (Bandura, 1997). Creative self-efficacy further points to the capability belief in completing creative tasks (Tierney and Farmer, 2002). In the music domain, self-efficacy is specifically manifested as students’ confidence in completing specific musical tasks (such as performance or composition) (Ritchie and Williamon, 2011), and recent research shows that combining self-efficacy cultivation with teaching reflection is significantly related to enhanced piano learning performance (Dong and Gedvilienė, 2025). When contextualized within music generative activities, this efficacy shapes how learners approach the risks and uncertainties inherent in creating original musical ideas (Burnard, 2012; Kaschub and Smith, 2009). Faced with open-ended piano composition tasks, students’ capability judgments may be related to their tendency to attempt, persist, and modify works (Zarza-Alzugaray et al., 2020). Constructivist practices provide space for autonomous choice and problem-solving, while reflective guidance helps students identify effective strategies and understand the modification process; both may co-occur with higher creative self-efficacy, which in turn may be associated with teacher-evaluated composition skills.

Self-determination theory posits that the needs for autonomy, competence, and relatedness are associated with motivation quality (Deci and Ryan, 2000; Bonneville-Roussy and Evans, 2025). In recent years, this theory has been widely applied in different music learning contexts, whether examining the sustained participation motivation of traditional instrument learners (Guan and Luo, 2025; Wieser and Müller, 2025), motivational characteristics in higher music education (Blanco-Novoa et al., 2023), or autonomy support in distance music teaching (Shaheen, 2022), all confirming the positive association between satisfying autonomy and competence needs on inducing intrinsic musical motivation. Within composition pedagogy, autonomy support is particularly vital because creative thinking in music necessitates learning environments that validate students’ independent musical decisions (Hickey, 2012; Webster, 2016). When students have appropriate choices, can handle tasks, and receive support, they are more likely to participate in composition based on interest and the satisfaction of the activity itself. The autonomous choices provided by constructivist practices and participation in composition intentions and evaluation criteria during reflective activities may both be related to intrinsic motivation for composition. Students with higher intrinsic motivation may be more willing to continuously generate, compare, and modify musical materials, thereby co-varying with composition fluency, work completeness, and expressive levels observed by teachers. This study measures intrinsic motivation for composition and does not equate it with the complete flow experience that includes dimensions like focused attention and altered sense of time. Based on the parallel indirect effects framework, this study proposes:

*H3*: Constructivist practices (CP) and reflective guidance (RG) are positively and indirectly associated with piano composition skills (CSC) via teacher-perceived student creative self-efficacy (CSE), respectively.

*H4*: Constructivist practices (CP) and reflective guidance (RG) are positively and indirectly associated with piano composition skills (CSC) via teacher-perceived student intrinsic motivation for composition (IM), respectively.

CSE and IM are included in the model as parallel mediating variables, without presupposing a sequential order between the two.

### 2.3 Moderating effect of composition teaching frequency

Composition teaching frequency reflects the frequency with which students are exposed to open musical tasks such as improvisation and composition. When such activities enter the classroom less frequently, constructivist practices may lack stable task carriers; when activities are more frequent, autonomous choice, scheme comparison, and context exploration can be reflected in more composition tasks. According to self-determination theory, sustained autonomy support may be more stably associated with students’ intrinsic participation than episodic autonomous experiences (Brandisauskiene et al., 2023). Therefore, the positive relationship between constructivist practices and intrinsic motivation for composition may strengthen as the frequency of composition teaching increases.

This study uses the frequency with which teachers arrange improvisation or composition activities as a moderating variable. This indicator only reflects the frequency of creative activities appearing in the classroom and does not represent teachers’ professional abilities, teaching quality, or comprehensive teaching experience. Accordingly, it is proposed:

*H5*: Composition teaching frequency moderates the association between constructivist practices (CP) and intrinsic motivation for composition (IM); compared to a lower composition teaching frequency, the positive association between CP and IM is stronger at a higher frequency.

Based on the above hypotheses, this study constructed a conceptual model among teaching practices, psychological states, and piano composition skills (see Figure 1).

**Figure 1:**
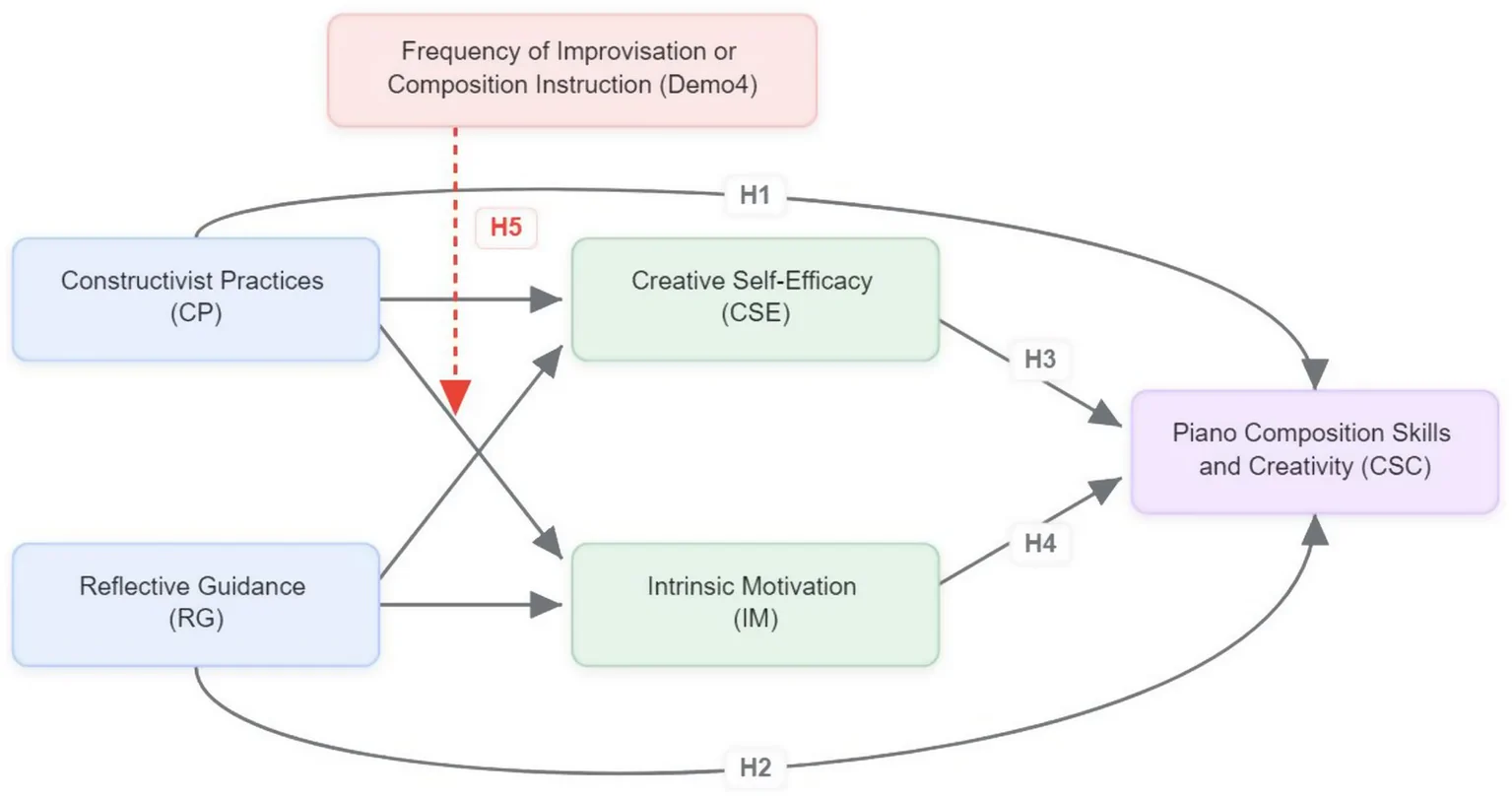
Conceptual model of teaching and psychological associations of adolescent piano composition skills. CP, constructivist practices; RG, reflective guidance; CSE, creative self-efficacy; IM, intrinsic motivation for composition; CSC, piano composition skills; Demo4, frequency of improvisation or composition teaching. Solid arrows indicate the direction of direct and parallel indirect hypotheses (H1–H4), while the dashed arrow (H5) illustrates the hypothesized moderating effect specifically applied to the CP → IM path.

## 3 Research methods

### 3.1 Sample and procedure

This study adopted a cross-sectional questionnaire survey design, with teachers having adolescent piano teaching experience as the survey subjects. The questionnaire was distributed through an online survey platform from March to April 2026, and disseminated via channels such as music education institutions, piano teacher professional communities, school music teaching and research networks, and personal studios. The survey scope was based in Gansu and Northwest China, and radiated to multiple provinces nationwide through the online network. Participants were required to be at least 18 years old, currently engaged in piano teaching or have continuous teaching experience within the year prior to the survey, teach subjects including adolescents, and be able to evaluate students’ improvisation, composition, or other creative musical activities based on actual teaching experience. Respondents who declined informed consent, failed the attention checks, or submitted incomplete core items were excluded from the analysis.

The first page of the questionnaire explained the research purpose, data usage, anonymity, voluntary participation principle, and the right to withdraw. After respondents confirmed informed consent digitally by affirmatively selecting the “Yes, I agree” option on the initial page, they entered the formal questionnaire. The study did not directly recruit minors, nor did it collect identifiable information from respondents or students. The data is only used for aggregate analysis and stored according to research ethics requirements.

A total of 800 questionnaires were collected in the survey. According to preset rules, records lacking informed consent confirmation, failing AC1, or having incomplete core items were excluded. A total of 723 valid questionnaires were finally retained, yielding an effective rate of 90.38%.

The background characteristics of the valid sample are shown in Table 1. The sample covers different teaching settings, teaching years, educational backgrounds, and composition teaching frequencies, among which teachers from social music training institutions account for a relatively high proportion. Due to the use of non-probability sampling, relevant proportions are only used to describe the sample of this study.

**Table 1:** Demographic and teaching background characteristics of the valid sample (*N* = 723).

Background characteristics	Code	Category	*n*	%
Main Teaching Setting (Demo1)	1	Professional Music Conservatories/Affiliated Middle Schools	76	10.5
	2	Comprehensive Schools/Music Departments of International Schools	108	14.9
	3	Social Music Training Institutions	387	53.5
	4	Personal Independent Studios	152	21.0
Actual Years of Music Teaching (Demo2)	1	Under 3 years	108	14.9
	2	3–7 years	294	40.7
	3	8–15 years	249	34.4
	4	Over 15 years	72	10.0
Highest Education Level (Demo3)	1	Associate Degree (College)	60	8.3
	2	Bachelor’s Degree	462	63.9
	3	Master’s Degree	192	26.6
	4	Doctoral Degree	9	1.2
Improvisation or Composition Teaching Frequency (Demo4)	1	Almost never	196	27.1
	2	Occasionally interleaved	294	40.7
	3	Frequently conducted	208	28.8
	4	Specialized in composition	25	3.5
Teacher-perceived Students’ Main Learning Purpose (Demo5)	1	Hobby/Interest	260	36.0
	2	Grading Tests or Competitions	431	59.6
	3	Professional Development	32	4.4

Percentages take the 723 valid questionnaires as the denominator and retain one decimal place. Due to rounding, the sum of category percentages may slightly deviate. Demo5 reflects teachers’ overall judgment of the main learning purpose of the adolescent students they teach, not students’ self-reports. Given that most teachers instruct mixed groups, this categorization captures the predominant educational orientation of their classes rather than assuming a uniform student profile.

### 3.2 Measurement tools

The questionnaire consisted of background information, five core construct scales, and attention screening questions. Background variables included main teaching setting (Demo1), years of teaching (Demo2), highest education level (Demo3), improvisation or composition teaching frequency (Demo4), and teacher-perceived students’ main learning purpose (Demo5). The five core constructs comprised a total of 19 items, all scored on a five-point Likert scale, with 1 indicating “*strongly disagree*” and 5 indicating “*strongly agree*.” The score for each construct was taken as the average of its constituent items; higher scores indicate that the corresponding characteristics perceived by teachers are more pronounced. Core items referenced the adolescent student group the teacher is currently or has recently been teaching continuously, requiring teachers to evaluate based on overall teaching observations rather than answering for a specific identifiable student. Therefore, CSE, IM, and CSC reflect teachers’ perceptions of the overall state of the students they teach. All items were positively scored. Details of the questionnaire and data codebook are provided in Supplementary Materials 1, 2.

Constructivist Practices (CP) contains 4 items, referencing the Constructivist Learning Environment Survey tool developed by Taylor et al. (1997), with phrasing adjusted according to the context of piano composition teaching. The items examine whether teachers allow students to choose composition themes or styles, apply music theory knowledge to composition tasks, compare different musical schemes, and participate in composition decision-making. This construct reflects teacher-reported constructivist teaching practices, not a complete replication of all dimensions of the original scale.

Reflective Guidance (RG) contains 4 items, referencing the reflective thinking measurement framework of Kember et al. (2000). The items examine whether teachers guide students to review the composition process, explain musical choices, identify problems in works, and participate in peer evaluation. Because the original scale mainly used self-reports by learners, this study measures teacher-reported reflective teaching activities, which is not equivalent to students’ own reflection levels.

Creative Self-Efficacy (CSE) contains 3 items, referencing the creative self-efficacy scale of Tierney and Farmer (2002). After contextual adjustment, the items evaluate whether teachers observe that students believe they can propose musical ideas, handle composition problems, and complete piano works. This construct reflects teachers’ perceptions of students’ creative capability beliefs.

Intrinsic Motivation (IM) contains 3 items, referencing the Intrinsic Motivation Inventory examined by McAuley et al. (1989). The items involve students’ interest, level of engagement when participating in piano composition, and their tendency to spontaneously participate in composition in the absence of external requirements like exams, competitions, or rewards.

Composition Skills and Creativity (CSC) contains 5 items, developed based on Amabile’s (1982) consensual assessment technique and Webster’s (1987) musical creative thinking measurement framework. The items involve the fluency and novelty of musical ideas, melodic and harmonic organization, work structure, modification ability, and expressive completeness. Since this study did not conduct independent expert scoring of students’ works, CSC represents teachers’ comprehensive evaluation of students’ composition skills, which is not equivalent to consensual assessment results based on actual works. Furthermore, this evaluation tends to examine students’ broad compositional ability to conceptualize and develop musical materials, rather than being limited only to spontaneously occurring and unmodifiable improvisational performances.

All items were contextually rephrased according to the specific context of adolescent piano teaching. Given that some reference scales were originally in English, this study strictly followed the standard “Translation and Back-translation” procedure: two researchers with good bilingual proficiency independently translated them into Chinese, and then another scholar who had not been exposed to the original English scales back-translated them, eliminating semantic deviations through comparison.

Subsequently, the initial Chinese draft was submitted to 3 experts in related fields (including 2 experts in higher education music education and piano teaching, and 1 expert in educational psychometrics) for review to ensure the content validity of each item and the clarity of expression. Before officially deploying the questionnaire, the researchers also conducted a small-scale pretest on 45 frontline adolescent piano teachers. Based on the pretest feedback, minor adjustments were made to certain expressions bearing traces of literal translation, making them fit more naturally into the specific context of domestic daily piano teaching. The Cronbach’s *α*, composite reliability (CR), and average variance extracted (AVE) of each scale are reported in the subsequent measurement model analysis.

### 3.3 Data analysis strategy

Data analysis was completed using IBM SPSS Statistics 26.0 and AMOS 24.0. SPSS was used for data screening, descriptive statistics, reliability analysis, Pearson correlation analysis, inter-group comparison, and regression path analysis based on construct mean scores; AMOS adopted the maximum likelihood method for confirmatory factor analysis. Harman’s single-factor test was used to initially diagnose common method bias (Harman, 1976). In addition, by introducing an unmeasured latent method construct (ULMC) in the confirmatory factor analysis, the potential bias from common method variance on the model structure was further controlled and tested (Podsakoff et al., 2003). The measurement model reported standardized factor loadings, Cronbach’s *α*, composite reliability (CR), average variance extracted (AVE), as well as *χ*^2^/*df*, CFI, TLI, RMSEA, and SRMR (Bagozzi and Yi, 1988).

Using the mean scores of the items belonging to each construct to form observed variables, multivariate regression path analysis was adopted to test the direct associations among CP, RG, and CSC, as well as the parallel indirect associations presented by CSE and IM. Indirect paths were estimated using 5,000 nonparametric Bootstrap resamplings, and the percentile method 95% confidence intervals were reported (Hayes, 2018). Given that the options of Demo4 (composition teaching frequency) reflect the progressive grading characteristics of teaching activities, this study treated it as a continuous variable reflecting frequency intensity. CP and Demo4 were standardized before constructing interaction terms to test the moderating role of composition teaching frequency on the association between CP and IM; the model simultaneously controlled for RG, main teaching setting, teaching years, highest education level, and students’ main learning purpose.

Differences among groups with different student learning purposes were tested using one-way ANOVA, and *η*^2^ was reported. All tests were two-tailed, and the significance level was set at *α* = 0.05. Given the cross-sectional design of the study, all paths are interpreted as concurrent statistical associations.

## 4 Research results

### 4.1 Measurement model and common method bias assessment

There was no missing data across the 19 core items for the 723 valid questionnaires, and values ranged from 1 to 5 points. Harman’s single-factor test extracted 5 components with eigenvalues greater than 1. The first component explained 34.24% of the total variance, showing no single dominant component. After introducing a model containing a global method factor, the model fit indices showed no substantive changes (e.g., ΔCFI < 0.01). This result served as a preliminary diagnosis supporting the subsequent analyses. The five-factor measurement model achieved an acceptable fit and was significantly superior to the three-factor and single-factor competing models, supporting the discriminant validity among CP, RG, CSE, IM, and CSC (see Table 2).

**Table 2:** Fit results of competing measurement models.

Model	*χ* ^2^	*df*	*χ*^2^/*df*	CFI	TLI	RMSEA	SRMR
Five-factor model	138.430	142	0.975	1.000^1^	1.000^1^	0.000	0.023
Three-factor model^2^	882.032	149	5.920	0.845	0.822	0.083	–
Single-factor model	1470.294	152	9.673	0.721	0.686	0.110	–

^1^Original calculated values were slightly above 1; they are reported as 1.000 based on the theoretical boundaries of fit indices. ^2^The three-factor model combined CP and RG into a teaching practice factor, CSE and IM into a psychological state factor, with CSC independently forming an outcome factor. “–” indicates not reported.

The internal consistency and composite reliability of all scales reached 0.70 or above, and the standardized factor loadings were all significant. The AVEs for CSE and IM were above 0.50; the AVEs for CP, RG, and CSC were slightly below 0.50, indicating that their convergent validity still needs further verification. Given that the composite reliability for all three was above 0.70, they were retained for subsequent analysis, but related results should be interpreted cautiously (see Table 3).

**Table 3:** Reliability, factor loadings, and convergent validity.

Construct	No. of Items	Cronbach’s *α*	Loading range	CR	AVE	Square root of AVE
CP	4	0.793	0.683—0.738	0.795	0.492	0.701
RG	4	0.793	0.652—0.723	0.794	0.491	0.701
CSE	3	0.755	0.685—0.748	0.756	0.508	0.713
IM	3	0.768	0.698—0.764	0.767	0.524	0.724
CSC	5	0.826	0.657—0.735	0.826	0.487	0.698

CR, composite reliability; AVE, average variance extracted. All standardized factor loadings reached statistical significance (*p* < 0.001).

The square root of the AVE for each construct was higher than its correlation coefficients with other constructs. Combined with the competing model results, the five-factor structure possesses acceptable discriminant validity.

### 4.2 Descriptive statistics, correlations, and inter-group differences

The means of the five constructs were all slightly above the scale midpoint, and there were moderate positive correlations among the constructs (see Table 4). Among them, the correlation coefficient between CSC and CSE was relatively high, but all correlations were below 0.50, showing no obvious construct overlap.

**Table 4:** Descriptive statistics and Pearson correlation coefficients of core constructs.

Construct	*M*	*SD*	1	2	3	4	5
1. CP	3.184	0.672	**0.701**				
2. RG	3.188	0.663	0.365***	**0.701**			
3. CSE	3.173	0.720	0.422***	0.416***	**0.713**		
4. IM	3.196	0.700	0.419***	0.342***	0.455***	**0.724**	
5. CSC	3.208	0.648	0.461***	0.420***	0.481***	0.450***	**0.698**

Bold values on the diagonal are the square roots of AVE; values below the diagonal are Pearson correlation coefficients. ***p < 0.001.

As shown in Table 5, overall differences among groups with different learning purposes on the five constructs all reached statistical significance. Post-hoc Bonferroni tests (adjusted for multiple comparisons) revealed that the mean scores of the grading test or competition group were significantly lower than those of the hobby group across all five constructs (CP: *MD* = −0.24, *p* < 0.001; RG: *MD* = −0.18, *p* = 0.002; CSE: *MD* = −0.21, *p* < 0.001; IM: *MD* = −0.22, *p* < 0.001; CSC: *MD* = −0.24, *p* < 0.001). Differences involving the professional development group (*n* = 32) mostly did not reach statistical significance after correction (e.g., versus the hobby group, all *p* > 0.05). Although its CP score was moderately higher than that of the grading group (*MD* = 0.40, *p* = 0.003), this professional group is too small to yield broadly stable estimates, and thus its descriptive trends should be interpreted with caution. Full details of all pairwise comparisons are provided in Supplementary Material 3: Table S2.

**Table 5:** Inter-group differences across different learning purposes.

Grouping variable	Construct	Group means	*F*	*p*	Effect size
Learning Purpose (Demo5)	CP	Hobby 3.32; Grading 3.08; Professional 3.48	*F* = 13.96	<0.001	*η*^2^ = 0.037
	RG	Hobby 3.30; Grading 3.12; Professional 3.20	*F* = 5.90	0.003	*η*^2^ = 0.016
	CSE	Hobby 3.30; Grading 3.09; Professional 3.30	*F* = 7.53	0.001	*η*^2^ = 0.020
	IM	Hobby 3.33; Grading 3.10; Professional 3.38	*F* = 9.58	<0.001	*η*^2^ = 0.026
	CSC	Hobby 3.35; Grading 3.11; Professional 3.35	*F* = 12.05	<0.001	*η*^2^ = 0.032

Sample sizes for Demo5 groups are 260 for the hobby group, 431 for the grading test or competition group, and 32 for the professional development group.

Additionally, an exploratory comparison of the core constructs across different teaching settings is provided in Supplementary Material 3: Table S1.

The mean distributions of the five core constructs under different learning purposes are shown in Figure 2. The means for the grading test or competition group are overall lower than those of the hobby group.

**Figure 2:**
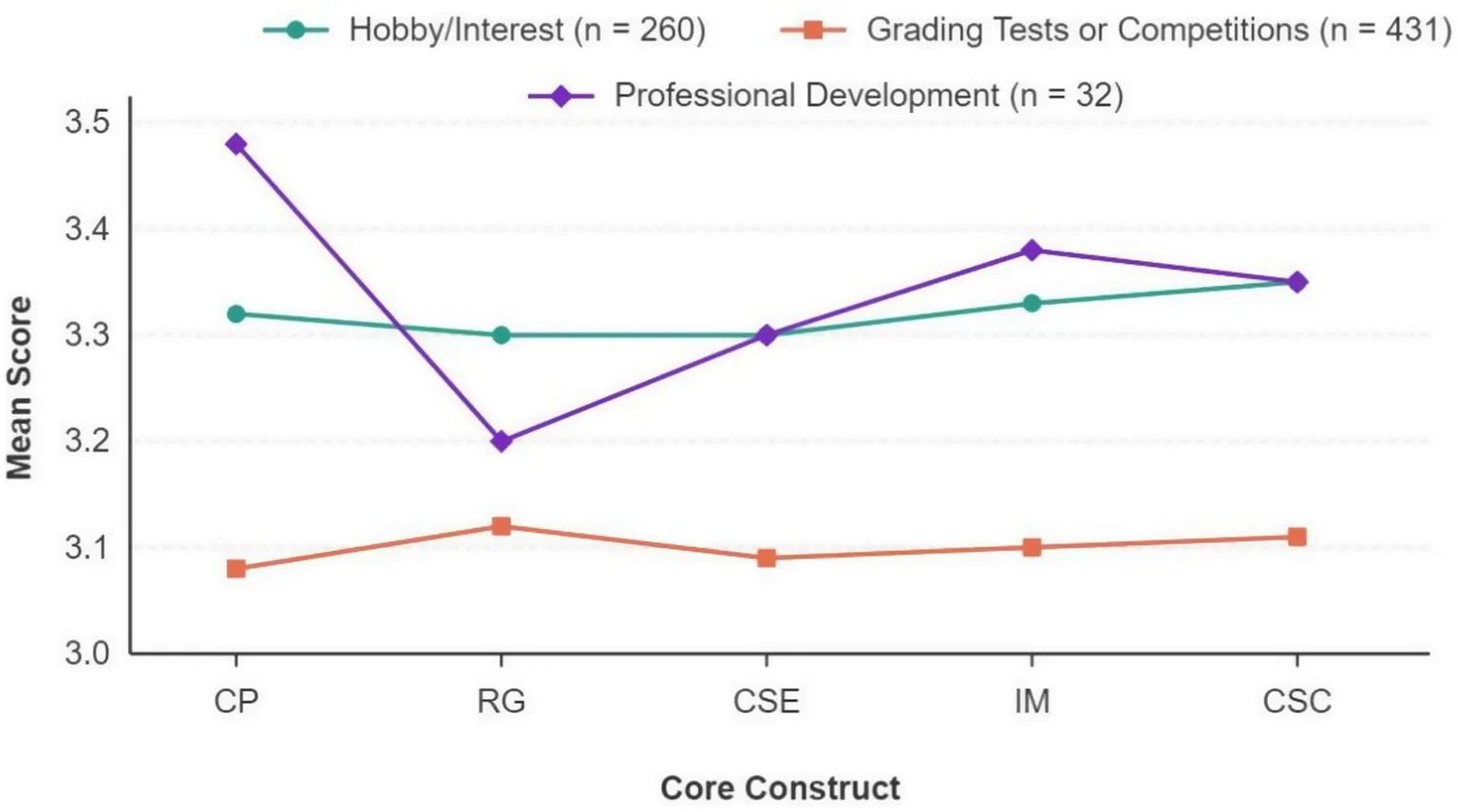
Mean distributions of core constructs under different learning purposes. The figure displays the construct means for the Hobby/Interest group, Grading Tests or Competitions group, and Professional Development group. CP, constructivist practices; RG, reflective guidance; CSE, creative self-efficacy; IM, intrinsic motivation for composition; CSC, piano composition skills.

### 4.3 Structural paths and parallel indirect effects

After controlling for Demo1–Demo5, CP and RG both showed significant positive associations with CSE, IM, and CSC, and the paths from CSE and IM to CSC also reached statistical significance, as shown in Table 6. Accordingly, H1 and H2 were supported. The models explained 27.3%, 23.2%, and 37.8% of the variance in CSE, IM, and CSC, respectively.

**Table 6:** Standardized path coefficients after controlling for background variables.

Outcome Variable	Predictor variable	*β*	*SE*	*p*	Model *R*^2^
CSE	CP	0.292	0.035	<0.001	0.273
	RG	0.291	0.035	<0.001	
IM	CP	0.319	0.036	<0.001	0.232
	RG	0.202	0.036	<0.001	
CSC	CP	0.210	0.035	<0.001	0.378
	RG	0.177	0.034	<0.001	
	CSE	0.220	0.036	<0.001	
	IM	0.186	0.035	<0.001	

The models control for Demo1–Demo5; Demo1 and Demo5 were dummy-coded as categorical variables. Coefficients in the table are standardized regression coefficients, and R2 indicates the proportion of variance explained by the model for the corresponding outcome variable.

After controlling for main teaching setting, teaching years, highest education level, composition teaching frequency, and students’ main learning purpose, both constructivist practices and reflective guidance showed significant positive associations with the two psychological variables and piano composition skills; creative self-efficacy and intrinsic motivation for composition were also, respectively, significantly and positively associated with piano composition skills. The standardized path coefficients and explained variance of the endogenous variables are shown in Figure 3.

**Figure 3:**
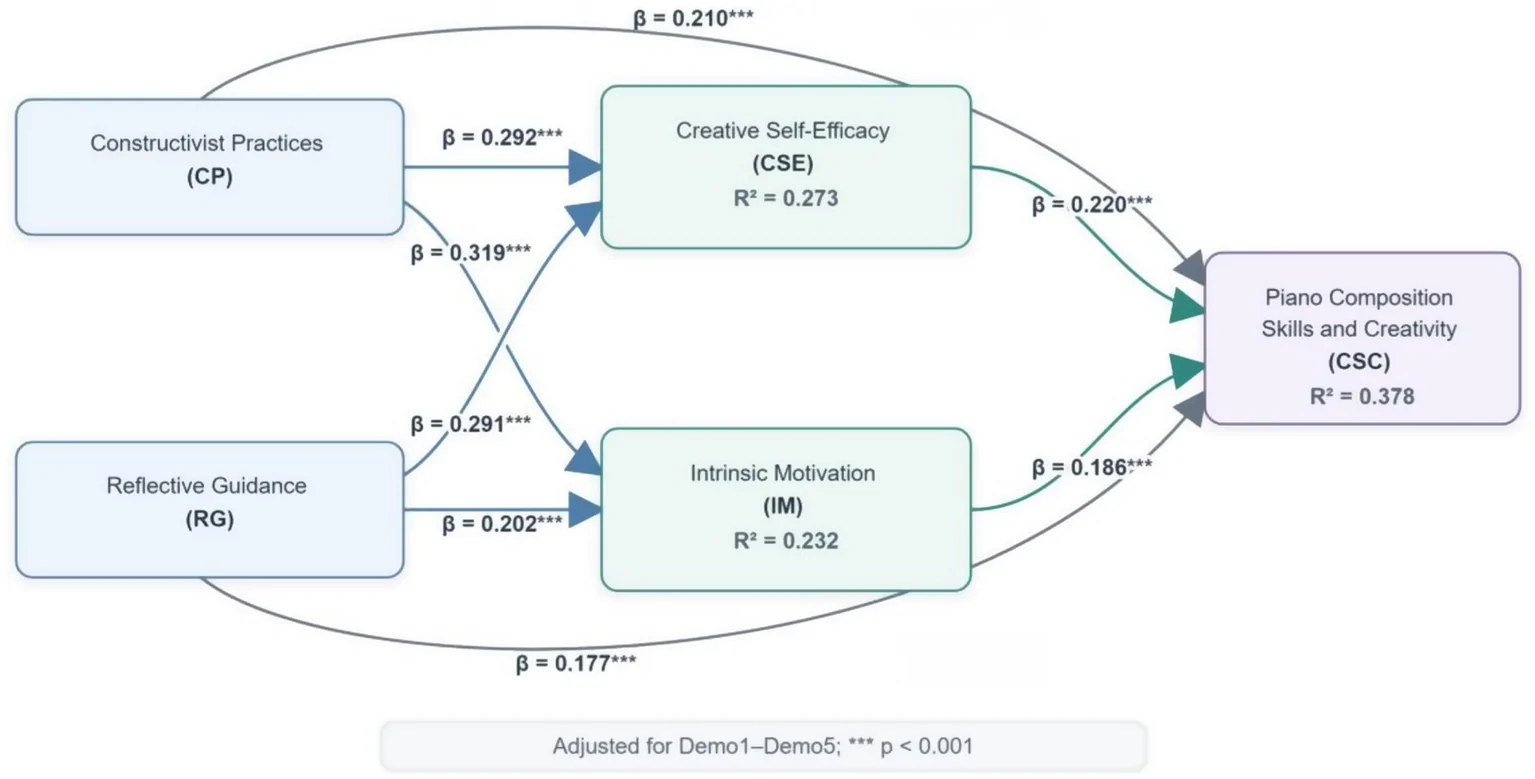
Empirical path model of teaching practices, psychological states, and piano composition skills. CP, constructivist practices; RG, reflective guidance; CSE, creative self-efficacy; IM, intrinsic motivation for composition; CSC, piano composition skills. Values in the figure are standardized regression coefficients, and *R*^2^ indicates the proportion of variance explained by the model for the endogenous variables. The model controls for Demo1–Demo5. ***p < 0.001.

Bootstrap tests showed that the 95% confidence intervals for the four indirect paths of CP and RG correlating with CSC via CSE and IM, respectively, did not include 0; the direct associations of CP and RG also remained significant, as shown in Table 7. The results supported H3 and H4, and presented a pattern where direct associations and parallel indirect associations co-exist.

**Table 7:** Bootstrap tests for parallel indirect paths.

Path	Standardized effect	95% CI lower limit	95% CI upper limit	Judgment
CP → CSE → CSC	0.064	0.040	0.092	Significant
CP → IM → CSC	0.059	0.036	0.086	Significant
CP Total indirect effect	0.124	0.092	0.159	Significant
CP → CSC direct effect	0.210	0.142	0.281	Significant
RG → CSE → CSC	0.064	0.041	0.090	Significant
RG → IM → CSC	0.038	0.021	0.057	Significant
RG total indirect effect	0.102	0.074	0.133	Significant
RG → CSC direct effect	0.177	0.112	0.241	Significant

Number of Bootstrap resamplings is 5,000. When the confidence interval does not include 0, the indirect path reaches statistical significance.

### 4.4 Moderating effect of composition teaching frequency

To test H5, standardized CP, composition teaching frequency, and their interaction term were included in the IM regression model, controlling for RG and other background variables. CP showed a significant positive association with IM, but neither the direct association of composition teaching frequency nor the CP × Demo4 interaction term reached statistical significance. The 95% confidence interval for the interaction term included 0; hence, there is no evidence indicating that the association between CP and IM significantly changes with composition teaching frequency. H5 was unsupported. Detailed regression results are in Table 8.

**Table 8:** Test of moderating role of composition teaching frequency.

Predictor variable	*β*	Robust *SE*	*p*	95% CI
CP	0.316	0.035	<0.001	0.247,0.386
Composition teaching frequency (Demo4)	−0.027	0.031	0.386	−0.089,0.034
CP × Demo4	−0.045	0.034	0.191	−0.112,0.022
RG	0.202	0.034	<0.001	0.135,0.269

Dependent variable is IM; *R*^2^ = 0.234. Continuous variables were standardized prior to constructing the interaction term, and standard errors were estimated using HC3 robust estimation. The model controls for Demo1, Demo2, Demo3, and Demo5.

To present the direction of the interactive relationship, a descriptive analysis of conditional slopes under different composition teaching frequencies was conducted. At a lower composition teaching frequency (−1SD), the conditional slope between CP and IM was 0.361; at a higher composition teaching frequency (+1SD), this slope was 0.271 (see Figure 4). Both slopes remained positive, but the direction of numerical change was not consistent with the “higher frequency, stronger positive association” expected in H5. Because the interaction term did not reach statistical significance and the confidence interval included 0, the aforementioned slope difference can only serve as a descriptive result and cannot be used to conclude that composition teaching frequency weakens or strengthens the association between CP and IM.

**Figure 4:**
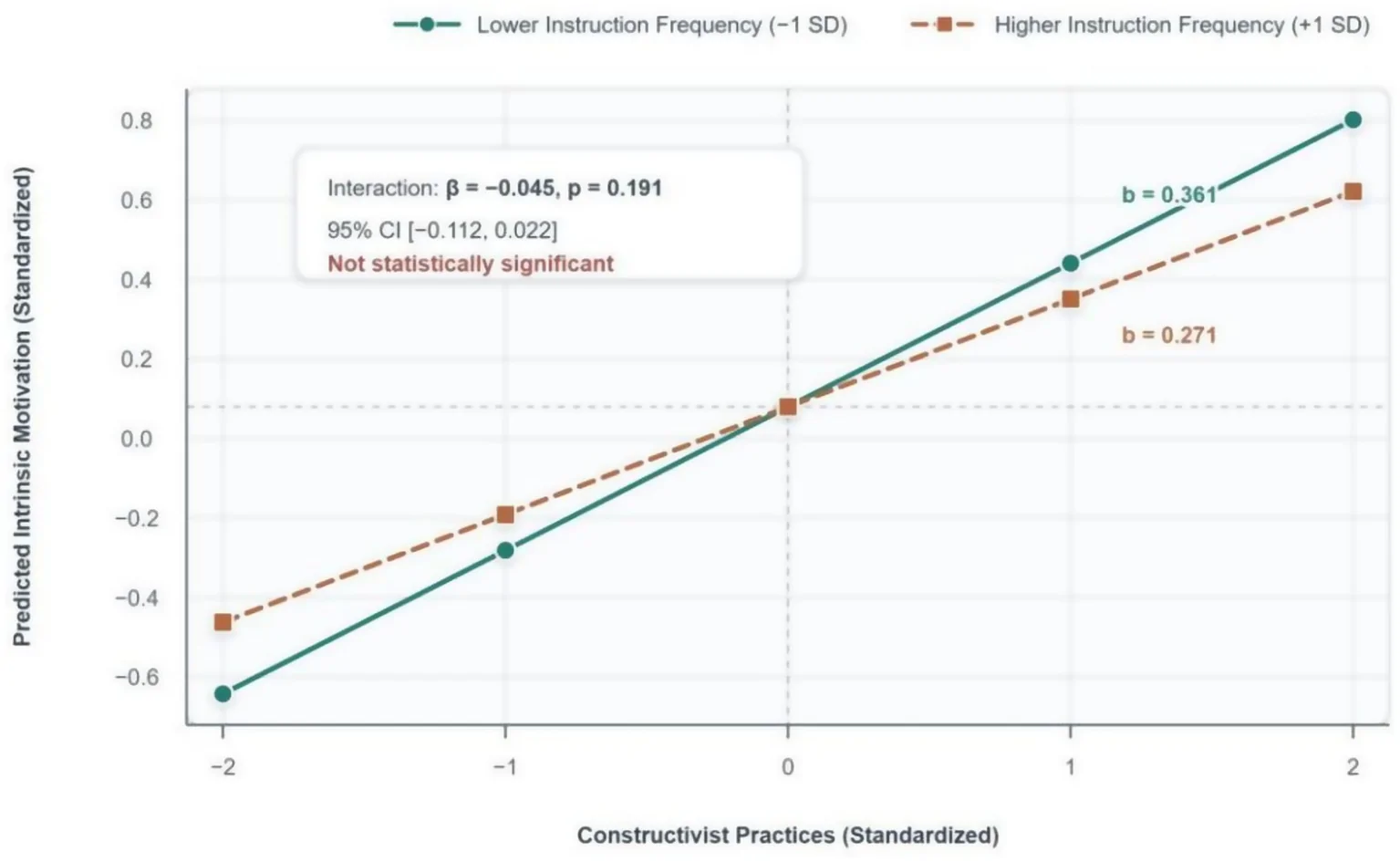
Conditional association between constructivist practices and intrinsic motivation for composition under different composition teaching frequencies. Lower and higher composition teaching frequencies represent 1 standard deviation below and above the mean, respectively. CP and Demo4 were standardized before constructing the interaction term. The model controls for RG, Demo1, Demo2, Demo3, and Demo5.

Current results did not provide evidence to view composition teaching frequency as a boundary condition for the CP–IM relationship. One possible explanation is that sheer activity frequency cannot reflect task openness, autonomy support, and feedback quality, but this explanation still awaits testing in subsequent research.

## 5 Discussion

### 5.1 Structural associations among teaching practices, psychological states, and composition skills

Constructivist practices and reflective guidance, respectively, correspond to two aspects of composition learning. The former involves students in selecting themes, materials, and expression methods, applying musical knowledge to specific compositional problems; the latter prompts students to scrutinize their compositional decisions through sight-reading, listening, explaining, and version comparison. Classroom openness cannot replace reflection, nor can reflective activities substitute students’ actual assumption of compositional responsibility. Both maintain independent associations with composition skill evaluation, indicating that autonomous decision-making and process scrutiny may be complementary teaching dimensions.

This result also helps in understanding the teacher’s role in the composition classroom. Learner-centeredness does not mean reducing teaching support, but rather adjusting the way support is given. Teachers no longer directly prescribe complete solutions, but help students handle the uncertainties in composition through task organization, problem prompts, and technical scaffolding, while reserving space for them to judge, compare, and revise their works. The resulting classroom structure balances openness with necessary constraints, enabling musical knowledge to be practically applied during the composition process.

The parallel indirect associations presented by creative self-efficacy and intrinsic motivation for composition provide a psychological explanation for the relationship between teaching practices and composition skills. Initial materials in composition are usually incomplete, and students need to tolerate temporary uncertainty and gradually form a work through sight-reading and revision. A classroom arrangement that accommodates exploration and adjustment may co-occur with students’ stronger capability beliefs. However, this study did not directly measure psychological safety or tolerance for errors, so a “safe space” cannot be considered an independently verified mechanism (Hendricks et al., 2024). Existing results can only indicate that the covariant structure among teaching practices, capability beliefs, and skill evaluations is consistent with relevant theoretical expectations. Given the cross-sectional nature of these observations, however, this structure represents concurrent associations rather than established causal mechanisms.

Intrinsic motivation reflects students’ interest, spontaneous participation, and engagement tendency in creative activities. Autonomous choice and expression of compositional intent connect tasks with students’ personal experiences, and may also make composition more than just an additional exercise under external requirements. Concurrently, higher motivation may accompany more compositional involvement, and existing skills might also make it easier for students to gain satisfaction from creative activities. Bidirectional associations may also exist among motivation, engagement, and skills. This study also did not measure flow dimensions such as challenge-skill balance, focused attention, and altered sense of time, thus not equating intrinsic motivation for composition with a flow experience.

### 5.2 Learning objectives, teaching settings, and composition teaching frequency

The descriptive means of the grading test or competition group on core constructs were overall lower than those of the hobby group, supporting observations that external evaluation structures may interact complexly with creative learning. Grading tests and competitions can provide stage goals, technical norms, and learning feedback. What needs attention is that when prescribed repertoire, performance accuracy, and result rankings become the primary basis for curriculum arrangements, experiments, pauses, and multi-scheme comparisons in composition tasks may struggle to find stable space. Therefore, inter-group differences are better understood as relationships worth further study among learning objectives, evaluation structures, and classroom resource allocation, rather than making a single value judgment about the grading test system.

External goals and creative learning are not mutually exclusive. Teachers can integrate motif continuation, harmonic substitution, textural reconstruction, and stylistic adaptation into the learning process of prescribed pieces, so that technical training simultaneously includes the analysis and application of musical materials. An interest orientation also does not inevitably correspond to open teaching; without appropriate task structures and process feedback, students’ original interests might not translate into sustained compositional participation. Thus, the role of learning objectives may depend on how they enter classroom evaluation and teaching organization, rather than just the objective category itself.

Differences among teaching settings also require cautious interpretation. Internal to social training institutions, the curriculum may center on grading test progress, or it might feature flexible compositional arrangements; schools and professional institutions similarly might be constrained by evaluation standards and curriculum capacity. Institutional categories are merely external markers of the educational ecology, and curriculum systems and teaching implementation methods within the settings still need further investigation.

Composition teaching frequency did not show the expected moderating role, which provides supplementary information for understanding classroom implementation conditions. The original hypothesis suggested that more frequent improvisation or composition activities could provide more stable task carriers for constructivist practices, but current results did not show that the association between CP and IM strengthens with increased frequency. This result does not mean that the frequency of creative activities lacks educational significance, but indicates that the number of activities might be insufficient to describe the implementation conditions of constructivist practices.

Frequently scheduling composition activities does not necessarily mean students have ample choice, nor does it guarantee tasks have appropriate difficulty, process feedback, and depth of reflection; relatively infrequent activities might also be thoroughly designed. Thus, “whether to conduct composition” and “how to conduct composition” in the classroom need to be distinguished. The metric for composition teaching frequency (Demo4) reflects the occurrence of activities and cannot substitute for teachers’ compositional experience, task openness, or feedback quality. The lack of support for H5 thus limits the interpretation of the quantitative dimension and also prompts subsequent research to separately measure the frequency and quality of composition teaching.

### 5.3 Practical implications

Based on the above association patterns, piano composition teaching can be adjusted from three aspects: task design, reflection organization, and feedback methods.

Composition tasks can reserve choice space for students within clear boundaries. Teachers can limit modes, rhythmic materials, musical form scales, or technical difficulty, while allowing students to decide on themes, musical imagery, and material combinations. Such designs can avoid the task ambiguity caused by complete openness, and also reduce the substitution of personal judgment associated with excessive prescriptions. For students encountering composition for the first time, tasks can start with rhythmic reorganization, melodic continuation, and accompaniment variation before gradually transitioning to more complete work organization.

Reflection should be embedded in the composition process, not just occur after a work is completed. Reflection-in-action can unfold through scheme sight-reading, auditory comparison, and intention explanation; reflection-on-action can be conducted via listening to recordings, composition logs, version comparisons, and peer feedback. Questions posed by teachers should preferably point to specific relationships between musical materials and expressions, such as whether melodic development maintains connection, and how harmonic changes affect tension, rather than merely judging the work as “good” or “bad.”

Feedback also needs to distinguish between the current state of a work and student capabilities (McPherson et al., 2022). Immature materials during composition belong to the scheme formation process and should not be immediately construed as a lack of capability. Teachers can explain the auditory effects produced by certain treatments, helping students identify existing strategies and adjustable aspects, while preserving their responsibility to decide the direction of revision. Providing a single answer too early might reduce students’ opportunities to compare schemes, while overly broad evaluations are hard to turn into actionable information.

For classrooms strongly oriented toward grading tests or competitions, composition activities need not be separated from technical training. Teachers can design motif continuation, harmonic substitution, textural reconstruction, and stylistic adaptation around prescribed works, allowing students to apply learned knowledge while analyzing pieces. Evaluation methods could also incorporate process materials like drafts, recorded versions, and reflective explanations, not solely relying on final works. Such arrangements can provide space for knowledge transfer and autonomous judgment while retaining technical requirements.

Teacher professional development should also not merely emphasize increasing the number of composition activities. Instead of simply maximizing the frequency of improvisation in the curriculum, educational administrators and frontline teachers should prioritize the depth of high-quality reflective dialogs and intentional task design. Training content can cover difficulty grading of open tasks, process questioning, discussions of student works. Schools and training institutions also need to provide a stable position for composition teaching in class hours and evaluation systems, avoiding reliance completely on the ad-hoc arrangements of individual teachers.

### 5.4 Research limitations and future directions

The cross-sectional design limits the temporal interpretation of the results. Teaching practices, students’ psychological states, and composition skill evaluations were all collected at the same time point, making it impossible to confirm the sequential order among variables; reverse relationships and omitted variables might also exist. Future research could employ multi-time-point longitudinal designs to, respectively, record changes in teaching practices, psychological states, and creative performance; curriculum intervention studies can be used to compare outcome differences under varying task structures and feedback methods. Additionally, limited by the nature of the data, this study set creative self-efficacy and intrinsic motivation for composition as parallel intermediary variables; however, strictly from a self-determination theory perspective, the establishment of competence beliefs often serves as a crucial precondition for internalizing and facilitating intrinsic motivation. Future research could adopt a cross-lagged panel model to further clarify the potential chain activation sequence between these two psychological variables, thereby empirically verifying their directional antecedents within adolescent creative musical learning.

A single information source might introduce common method bias and halo biases. Teachers simultaneously evaluated their own teaching practices as well as students’ psychological states and composition skills, and their overall impression might affect ratings across multiple constructs. Although prior statistical tests indicated that method variance did not dominate the model results, subjective evaluations carrying social desirability could still somewhat strengthen the associations between teaching practices and student performance. For example, teachers who perceive themselves as providing high autonomy support might also be more inclined to perceive students’ intrinsic motivation and compositional highlights. Therefore, subsequent research could employ teacher-student paired reports, where teachers evaluate teaching practices, students report their own efficacy and motivation, and independent reviewers assess creative performance based on anonymous works, using multi-source interactions to further validate this study’s findings.

Measurement tools still require further validation. CSC represents teachers’ comprehensive evaluation of students’ composition skills, which is not equivalent to consensual assessment of actual works; the AVEs of CP, RG, and CSC were slightly below 0.50, indicating room for improvement in related contextualized scales. Future studies can combine cognitive interviews, standardized composition tasks, reviewer agreement analysis, and measurement invariance testing to re-examine item content and construct structure.

Composition teaching frequency was measured using a single ordinal item, unable to cover activity duration, task openness, feedback quality, and teachers’ composition teaching experience. This operationalization might have limited the identification of moderating relationships. Subsequent research could separately measure activity frequency and implementation quality, treating task structure, teacher support methods, and students’ existing compositional experience as possible boundary conditions. If moderating relationships are still not found under different designs, the association strength between CP and IM might decouple from activity frequency itself, suggesting that subsequent research should shift focus to investigating specific classroom interaction quality and task suitability.

Non-probability sampling also limits the generalizability of the results. The sample in this study had a high proportion of teachers from social music training institutions, and related findings cannot be directly generalized to all piano teachers or adolescent students. Future studies could expand regional coverage and employ stratified sampling; if the data contain hierarchical structures where multiple students belong to the same teacher or institution, multilevel models should also be used to distinguish differences at the individual, teacher, and institutional levels.

## 6 Conclusion

Based on teacher reports, this study found that both constructivist practices and reflective guidance are positively associated with adolescent piano composition skills, and creative self-efficacy and intrinsic motivation for composition present significant indirect associations, respectively. After incorporating the two psychological variables, the direct associations between constructivist practices, reflective guidance, and composition skills still reached statistical significance, indicating that teaching practices and psychological states provide independent explanatory information, respectively; demonstrating that autonomous decision-making, process reflection, and students’ psychological states jointly constitute relevant conditions for evaluating composition skills.

Composition teaching frequency did not significantly moderate the association between constructivist practices and intrinsic motivation for composition. This result does not negate the significance of introducing creative activities into everyday classrooms, but suggests that the number of activities cannot substitute for task design, feedback quality, and ways of student participation. Beyond technical training and repertoire learning, adolescent piano education can reserve a stable space for musical material exploration, autonomous judgment, process reflection, and work revision, while avoiding simplifying composition teaching into frequency metrics.

The above findings are limited to concurrent associations at the teacher report level, and all identified relationships represent statistical covariation rather than causal pathways. Future studies can combine teacher-student pairings, blind reviews of works, and longitudinal data to test their stability and contextual scope of application.

## Data Availability

The original contributions presented in the study are included in the article/Supplementary material, further inquiries can be directed to the corresponding author.
