# Supplementary material for: Teaching and psychological associations of adolescent piano composition skills: a study on constructivist practices and reflective guidance based on teacher reports
Source: Front Psychol. 2026 Oct 16;17:1963250. doi: 10.3389/fpsyg.2026.1963250 (PMC13719563; doi:10.3389/fpsyg.2026.1963250)
Supplement: Supplementary file 1 [file Data_Sheet_1.zip › Supplementary_Data_Sheet_2_Data_Codebook.docx]

Supplementary Material 2: Data Codebook (数据编码本)

Title: Teaching and Psychological Associations of Adolescent Piano Composition Skills: A Study on Constructivist Practices and Reflective Guidance Based on Teacher Reports

Overview: This codebook outlines the variables, labels, and coding schemes used for data entry and statistical analysis based on the survey instrument provided in Supplementary Material 1.

1. General Information & Demographics (基本信息与控制变量)

| Variable Name (变量名) | Variable Label (变量标签) | Description / Item Text (描述/题干摘要) | Values and Coding Scheme (赋值与编码说明) | Data Type (数据类型) |
| --- | --- | --- | --- | --- |
| ID | Participant ID | Unique identifier assigned to each respondent. | 1, 2, 3… (Auto-generated numbering) | Numeric |
| Consent | Informed Consent | Voluntarily agree to participate. | 1 = Yes (Included in dataset) Note: “No” responses terminated the survey and are excluded. | Nominal |
| Demo1 | Teaching Environment | Primary Teaching Environment (主要教学场域) | 1 = Conservatory/Affiliated School (专业院校) 2 = General/Intl School (综合/国际学校) 3 = Private Institution (社会机构) 4 = Independent Studio (独立工作室) | Nominal |
| Demo2 | Teaching Experience | Years of Music Teaching Experience (教龄) | 1 = < 3 years 2 = 3-7 years 3 = 8-15 years 4 = > 15 years | Ordinal |
| Demo3 | Education Level | Highest Educational Attainment (最高学历) | 1 = Associate Degree (专科) 2 = Bachelor’s Degree (本科) 3 = Master’s Degree (硕士) 4 = Doctorate (博士) | Ordinal |
| Demo4 | Improv Frequency | Frequency of incorporating improvisation (即兴教学频率) | 1 = Rarely (几乎没有) 2 = Occasionally (偶尔穿插) 3 = Frequently (经常进行) 4 = Exclusively (专注于创作) | Ordinal |
| Demo5 | Primary Motivation | Primary motivation of adolescent students (学生主要动机) | 1 = Leisure/Interest (兴趣爱好) 2 = Exams/Competitions (考级比赛) 3 = Professional Career (走专业道路) | Nominal |

**2. Main Measurement Scales (核心量表变量)**

All items in this section are measured on a 5-point Likert scale. (本部分所有题项均采用5点李克特量表赋值)

Coding Rule: 1 = Strongly Disagree, 2 = Disagree, 3 = Neutral, 4 = Agree, 5 = Strongly Agree.

| Variable Name (变量名) | Construct (所属构念) | Description / Key Concept (变量核心含义) | Required Action / Notes (数据处理备注) |
| --- | --- | --- | --- |
| CP1 | Constructivist Practice | Explore composition based on preferred styles. | Normal coding (1-5) |
| CP2 | Constructivist Practice | Guide students to try various solutions themselves. | Normal coding (1-5) |
| CP3 | Constructivist Practice | Allow autonomy into theme/length decisions. | Normal coding (1-5) |
| CP4 | Constructivist Practice | Contextualize music theory in authentic scenarios. | Normal coding (1-5) |
| CP_Mean | Constructivist Practice | Mean score of CP1 to CP4 (建构主义实践均值) | computed: MEAN(CP1, CP2, CP3, CP4) |
| RG1 | Reflective Guidance | verbalize intuitive auditory feelings (Reflection-in-action). | Normal coding (1-5) |
| RG2 | Reflective Guidance | Actively summarize the process after completion. | Normal coding (1-5) |
| RG3 | Reflective Guidance | Explain reasons for specific pedal/texture use. | Normal coding (1-5) |
| RG4 | Reflective Guidance | Organize peer-review sessions. | Normal coding (1-5) |
| RG_Mean | Reflective Guidance | Mean score of RG1 to RG4 (反思性引导均值) | computed: MEAN(RG1, RG2, RG3, RG4) |
| CSE1 | Creative Self-Efficacy | Less anxiety when faced with a blank score. | Normal coding (1-5) |
| CSE2 | Creative Self-Efficacy | Believe in capability to produce unique excerpts. | Normal coding (1-5) |
| CSE3 | Creative Self-Efficacy | Higher self-efficacy in challenging tasks. | Normal coding (1-5) |
| AC1 | Attention Check | To ensure data quality, must select “Agree” (4). | Data Cleaning: If AC1 ≠ 4, delete case. |
| CSE_Mean | Creative Self-Efficacy | Mean score of CSE1 to CSE3 (自我效能感均值) | computed: MEAN(CSE1, CSE2, CSE3) |
| IM1 | Intrinsic Motivation | Intense concentration / entering flow state. | Normal coding (1-5) |
| IM2 | Intrinsic Motivation | Spontaneously write melodies out of interest. | Normal coding (1-5) |
| IM3 | Intrinsic Motivation | View bottleneck as driver to perfect work. | Normal coding (1-5) |
| IM_Mean | Intrinsic Motivation | Mean score of IM1 to IM3 (内在动机均值) | computed: MEAN(IM1, IM2, IM3) |
| CSC1 | Composition Skills | Fluency (快速延展动机) | Normal coding (1-5) |
| CSC2 | Composition Skills | Novelty (打破常规的元素) | Normal coding (1-5) |
| CSC3 | Composition Skills | Structural Integrity (独立组织曲式) | Normal coding (1-5) |
| CSC4 | Composition Skills | Aesthetic Expression (音色与表达统一) | Normal coding (1-5) |
| CSC5 | Composition Skills | Overall Assessment (综合提升显著) | Normal coding (1-5) |
| CSC_Mean | Composition Skills | Mean score of CSC1 to CSC5 (创作技能均值) | computed: MEAN(CSC1...CSC5) |
