# Supplementary material for: Teaching and psychological associations of adolescent piano composition skills: a study on constructivist practices and reflective guidance based on teacher reports
Source: Front Psychol. 2026 Oct 16;17:1963250. doi: 10.3389/fpsyg.2026.1963250 (PMC13719563; doi:10.3389/fpsyg.2026.1963250)
Supplement: Supplementary file 1 [file Data_Sheet_1.zip › Supplementary_Data_Sheet_1_Survey_Instrument.docx]

**Supplementary Material 1: Survey Instrument (Bilingual Version)**

Title: Teaching and Psychological Associations of Adolescent Piano Composition Skills: A Study on Constructivist Practices and Reflective Guidance Based on Teacher Reports

Note: The original survey was administered in Chinese. An English translation is provided below for reference. The questionnaire utilizes a 5-point Likert scale (1 = Strongly Disagree / 完全不符合, 5 = Strongly Agree / 完全符合) for all main constructs.

**Part I: Informed Consent (知情同意书)**

[English] Dear Music Educator: This questionnaire is part of an academic survey on music education methods, aiming to understand how current piano teaching practices foster musical creativity and composition skills in adolescents (ages 12-18) from the professional perspective of frontline teachers. Your responses will be completely anonymous and used solely for statistical analysis and academic research. Completion takes approximately 5-8 minutes, and you may withdraw at any time. Thank you for your professional support!

[Chinese] 尊敬的音乐教育工作者：您好！本问卷属于一项关于音乐教育方法的学术调查，旨在通过一线教师的专业视角，了解当前的钢琴教学实践如何促进青少年（约12-18岁段）音乐创造力与作曲技能的发展。您的回答将采取完全匿名方式处理，仅用于整体的统计学分析与学术研究。完成问卷大约需要5-8分钟，您随时有权中止答题。感谢您的专业支持！

Consent to Participate (参与意愿确认):

Have you read the information above and do you voluntarily agree to participate in this study? (您是否已阅读上述说明，并自愿同意参与本项研究？)

[ ] Yes, I agree. (Proceed to the questionnaire)

是，我同意参与。 (进入下一页继续答题)

[ ] No, I do not agree. (Terminate the survey immediately)

否，我不同意。 (直接结束退出问卷)

**Part II: Demographic and Teaching Background (控制变量)**

Please select the option that best matches your situation. (请根据实际情况进行选择。)

**Demo1. Primary Teaching Environment (主要教学场域):**

[ ] Professional Music Conservatory/Affiliated School (专业音乐院校/附中)

[ ] General/International School Music Department (综合/国际学校音乐部)

[ ] Private Music Training Institution (社会音乐培训机构)

[ ] Independent Music Studio (个人独立工作室)

**Demo2. Years of Music Teaching Experience (音乐教学实际教龄):**

[ ] < 3 years (3年以下) [ ] 3-7 years (3-7年) [ ] 8-15 years (8-15年) [ ] > 15 years (15年以上)

**Demo3. Highest Educational Attainment (最高学历):**

[ ] Associate Degree (专科) [ ] Bachelor’s Degree (本科) [ ] Master’s Degree (硕士) [ ] Doctorate (博士)

**Demo4. Frequency of incorporating “improvisation/composition” in adolescent lessons (涉及“即兴/作曲”教学的频率):**

[ ] Rarely (几乎没有) [ ] Occasionally (偶尔穿插) [ ] Frequently (经常进行) [ ] Exclusively focusing on composition (专注于创作教学)

**Demo5. According to your observation, the primary motivation for most of your adolescent students to learn piano is (根据您的观察，您所教授的青少年学生，其学习钢琴的主要内驱力/目的多为):**

[ ] Leisure, interest, and comprehensive accomplishment (纯粹的兴趣爱好与综合素养培养)

[ ] Passing grading exams or internal competitions (为了通过各类钢琴考级或应付比赛)

[ ] Pursuing a professional music career (走向专业音乐院校/艺考道路)

**Part III: Main Measurement Scales (核心测量量表)**

Instruction: Based on your long-term observations of teaching piano composition to adolescents (12-18), please rate the following statements on a scale of 1 to 5. (说明：以下题目请基于您在“教授12-18岁青少年进行钢琴创作/即兴”时的长期观察进行评价。)

(1 = Strongly Disagree/完全不符合, 2 = Disagree/比较不符合, 3 = Neutral/一般或不确定, 4 = Agree/比较符合, 5 = Strongly Agree/完全符合)

**Scale 1: Constructivist Practice (建构主义实践, CP)**

Adapted from the Constructivist Learning Environment Survey (CLES).

CP1: I encourage students to explore composition based on their preferred musical styles rather than solely assigning fixed formal tasks. (在创作教学中，我倾向于鼓励学生基于自己偏好的音乐风格进行探索，而不是仅仅布置固定的曲式任务。)

CP2: When a student struggles with melody or harmony, I guide them to try various solutions themselves instead of directly providing the “correct answer.” (当学生构思遇到困难时，我通常引导他们自己尝试不同的解决方案，而不是直接给出“标准答案”。)

CP3: I allow adolescent students a degree of autonomy to decide the theme, emotional tone, or length of their compositions. (我允许青少年学生在一定范围内，自主决定他们作品的主题、情感基调或篇幅长短。)

CP4: I contextualize music theory (e.g., chord progressions) within authentic compositional scenarios that students are currently experiencing. (我会将乐理知识与学生正在经历的真实创作情境结合起来，让他们感受到知识的实用性。)

**Scale 2: Reflective Guidance (反思性引导效用, RG)**

Adapted from the Questionnaire on Reflective Practice (QRP).

RG1: After a student plays an improvised motif, I frequently ask them to immediately verbalize their intuitive auditory feelings (Reflection-in-action). (在学生弹奏完一段即兴动机后，我经常要求他们立刻口头描述自己听到该段音乐的直观感受。)

RG2: Upon completing a piece, I require students to actively summarize the process (e.g., identifying the most satisfying parts and areas for revision). (完成阶段性作品后，我会要求学生主动作总结，如最满意的部分和需修改之处。)

RG3: I observe that asking students to explain “why they used a specific pedal/texture” significantly deepens their understanding of musical structure. (我观察到，要求学生解释“为何使用特定踏板/织体”能明显加深其对音乐结构的理解。)

RG4: I organize peer-review sessions for compositions and find them irreplaceable in helping students refine their creative thinking. (我会组织同伴间的作品互评，我认为这对帮助学生修正后续创作思路有不可替代的作用。)

**Scale 3: Creative Self-Efficacy (青少年创造力自我效能感, CSE)**

Adapted from Tierney & Farmer (2002).

CSE1: When faced with a new, blank score requiring improvisation, students show notably less anxiety and apprehension. (面对一段需要即兴编配的全新空白谱面时，学生表现出的畏难与焦虑情绪明显减少。)

CSE2: Students increasingly believe in their own capability to produce “unique” and “aesthetically pleasing” musical excerpts. (学生越来越坚信自己有能力写出“与众不同”且“好听”的音乐片段。)

CSE3: When confronted with challenging compositional tasks (e.g., variations, complex harmony), students exhibit higher self-efficacy, believing they will ultimately succeed. (在面对变奏、复杂和声等挑战性任务时，学生展现出更强的自我效能感，相信自己能攻克难题。)

AC1 (Attention Check / 注意力筛查题): To ensure data quality, please select “Agree” for this item. (为了确保数据质量，请您在这一题直接选择“比较符合/4分”。) (注: 选其他选项的问卷将被视为无效数据)

**Scale 4: Intrinsic Motivation for Composition (青少年内在创作动机, IM)**

Adapted from the Intrinsic Motivation Inventory (IMI).

IM1: During the composition process, students often exhibit intense concentration, sometimes losing track of time (entering a flow state). (学生在钢琴创作过程中经常会表现出强烈的专注感，有时甚至会忘记时间，进入心流状态。)

IM2: Even without the pressure of exams or competitions, students spontaneously experiment with writing melodies at home out of pure interest. (哪怕没有考级或比赛压力，学生也会出于纯粹的爱好而在家尝试自己写旋律。)

IM3: When hitting a compositional bottleneck or receiving constructive criticism, they view it as an intrinsic driver to perfect their work rather than a failure. (当创作遇到瓶颈或被指出不足时，他们不再将其视为失败，而是将其视作完善作品的内在驱动力。)

**Scale 5: Composition Skills & Creativity (钢琴创作技能与创造力评估, CSC)**

Synthesized from Webster’s Measure of Creative Thinking in Music (MCTM) and Consensual Assessment Technique (CAT).

CSC1 (Fluency / 流畅性): Students can rapidly and fluently develop a complete phrase based on a simple musical motif. (学生能够根据一个简单的音乐动机，快速、流畅地延展出完整的乐句。)

CSC2 (Novelty / 新颖性): Students’ compositions frequently feature unexpected, unconventional musical elements that remain aurally coherent. (学生的作曲中经常会出现打破常规、但在听觉上依然成立的音乐元素。)

CSC3 (Structural Integrity / 结构完整): Students demonstrate sound musical logic, capable of independently structuring forms (e.g., A-B-A ternary form) with clear development and resolution. (学生具备良好的音乐逻辑，能够独立组织完整曲式，使整体听起来具有发展和回归。)

CSC4 (Aesthetic Expression / 审美表达): Students’ control of keyboard timbre highly aligns with their creative intentions, delivering strong artistic emotional impact. (学生的键盘音色控制与创作意图高度统一，具备强烈的艺术感染力。)

CSC5 (Overall Assessment / 综合评价): Compared to rote-learning peers, students trained under constructivist reflection show significantly more robust advancements in comprehensive compositional skills. (综合来看，经过建构反思训练的学生，其作曲技能的提升比接受传统机械教学的学生更为显著和扎实。)
